# Host‐induced gene silencing of multiple genes of *Fusarium graminearum* enhances resistance to Fusarium head blight in wheat

**DOI:** 10.1111/pbi.13401

**Published:** 2020-06-11

**Authors:** Minhui Wang, Lei Wu, Yuzhen Mei, Youfu Zhao, Zhonghua Ma, Xu Zhang, Yun Chen

**Affiliations:** ^1^ Key Laboratory of Molecular Biology of Crop Pathogens and Insects Institute of Biotechnology Zhejiang University Hangzhou China; ^2^ State Key Laboratory of Rice Biology Institute of Biotechnology Zhejiang University Hangzhou China; ^3^ Provincial Key Laboratory of Agrobiology Institute of Food Crops Jiangsu Academy of Agricultural Sciences Nanjing China; ^4^ Department of Crop Sciences University of Illinois at Urbana‐Champaign Urbana IL USA

**Keywords:** host‐induced gene silencing, wheat, *Fusarium graminearum*, deoxynivalenol, virulence, siRNA

Fusarium head blight (FHB) caused predominately by *Fusarium graminearum* complex is a devastating disease of wheat. Frequent epidemics of FHB have been reported in all FHB‐prone regions during the last decade, especially in China. FHB not only causes the loss of grain yield, but also raises food safety risks due to the harmful mycotoxin contamination in infected grains. Deoxynivalenol (DON) is the most common and economically important Fusarium mycotoxin identified in cereal grains, and it is also a critical virulence factor for *F. graminearum* infection (Chen *et al*., 2019). Resistant varieties are the most economical approach in controlling FHB. However, sources for FHB resistance are limited and resistant wheat cultivars are often accompanied by undesired agronomic traits, making conventional disease‐resistance breeding difficult (Bai and Shaner, 2004). Recently, a RNA interference (RNAi)‐based approach called host‐induced gene silencing (HIGS) has been developed to control fungal diseases, where small interference RNAs (siRNAs) that match important genes of the invading pathogen are produced by transgenic host plants to silence fungal genes during infection (Machado *et al*., 2018). The use of HIGS to control *F. graminearum* was first demonstrated by Koch et al., in which detached leaves of both transgenic *Arabidopsis* and barley plants expressing double‐stranded RNA (dsRNA) from cytochrome P450 lanosterol C‐14α‐demethylase genes exhibit elevated resistance to *F. graminearum* (Koch *et al*., 2013). However, detached leaves might not represent natural *F. graminearum* floral infections. In addition, the HIGS transgenic wheat targeting the chitin synthase 3b also confers resistance to FHB (Cheng *et al*., 2015). These data suggest that the HIGS strategy is valuable for developing FHB‐resistant wheat cultivars. However, DON is a secondary metabolite, and there are few reports on utilization of the HIGS technology for management of secondary metabolism. In this study, we evaluated the potential of HIGS for development of transgenic wheat plants against both FHB and DON contamination by simultaneously silencing three genes of *F. graminearum*.

Target gene selection is the first and crucial step for HIGS. Here, *FgSGE1* (also named as *FGP1*, locus FGSG_12164) encoding a critical regulator controlling DON biosynthesis (Jonkers *et al*., 2012), *FgSTE12* (locus FGSG_07310) encoding a key transcriptional factor for penetration structure formation *in planta* (Gu *et al*., 2015) and *FgPP1* (locus FGSG_07233) encoding an essential phosphatase (Yun *et al*., 2015) were selected as target genes for HIGS. To design a chimeric hairpin RNAi construct that could simultaneously silence three target genes (Figure 1a), a tandem DNA fragment containing a 243‐bp partial *FgSGE1‐*coding region, a 233‐bp partial *FgSTE12‐*coding region and a 224‐bp partial *FgPP1‐*coding region (Figure 1b) was constructed and inserted into pSilent‐1 vector to generate a dsRNA sequence with a hairpin structure. The RNAi cassettes were highly specific to the target *F. graminearum* genes with no consecutive 21 to 24 nucleotide sequences found in wheat and human genomes by BLASTN search. The chimeric RNAi construct was first transformed into the wild‐type *F. graminearum* strain PH‐1 to test the efficiency in silencing corresponding target genes in fungus. Four out of 16 resulting transformants showed multi‐phenotypic changes, compared with these in PH‐1, indicating that effective silencing of target genes did occur. The transformant designated as FgSSP‐S4 exhibited similar phenotypes as deletion mutants of three target genes, that is, slower growth rate (silencing of *FgPP1*), the reduced DON production (silencing of *FgSGE1*) and the decrease of infection structures on wheat lemma (silencing of *FgSTE12*) (Figure 1c). Consistent with various phenotypic defects in FgSSP‐S4, expression levels of the three target genes were significantly decreased *in vitro*, as compared to those in PH‐1 (Figure 1d). These results indicate that *FgSGE1*‐*STE12*‐*PP1* RNAi construct is able to effectively and simultaneously silence the three target genes in *F. graminearum*, which inspired us to develop FHB‐resistant wheat through the HIGS technology.

**Figure 1:**
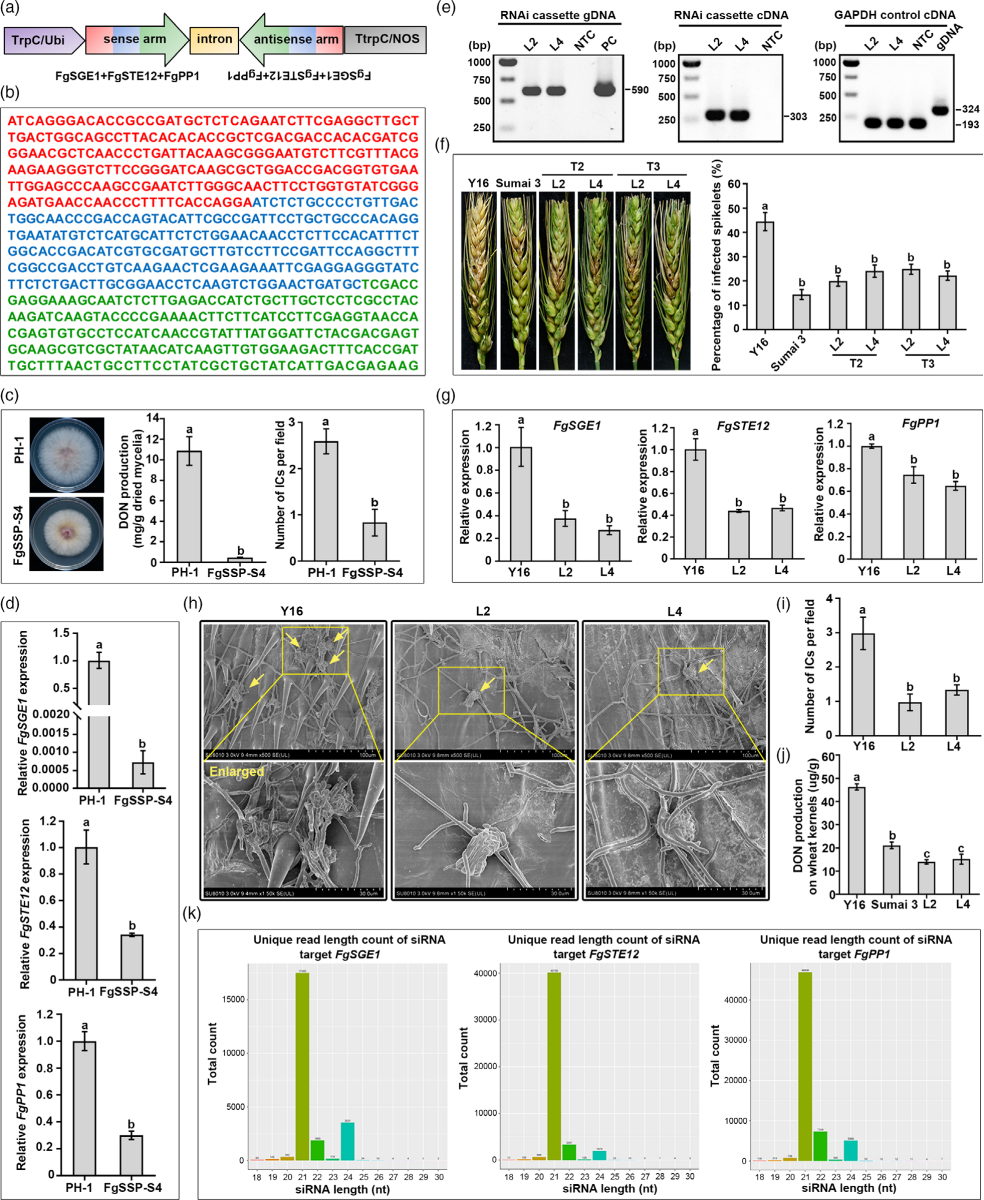
HIGS of chimeric *FgSGE1*‐*STE12*‐*PP1* results in strong resistance to Fusarium head blight. (a) Schematic map of the *FgSGE1*‐*STE12*‐*PP1* RNAi cassettes. Trp*C*, fungus promoter; Ttrp*C*, fungus terminator; Ubi, plant promoter; NOS, plant terminator. (b) Sequence representation of *FgSGE1*‐*STE12*‐*PP1* RNAi fragments. *FgSGE1*, *FgSTE12* and *FgPP1* are indicated in red, blue and green, respectively. (c) Silencing transformant FgSSP‐S4 reduces mycelial growth, DON production in DON inducing medium (TBI) at 7 days post‐inoculation (dpi) and infection cushions (ICs) on wheat lemma at 3 dpi. (d) Relative expression levels of *FgSGE1*, *FgSTE12* and *FgPP1* are decreased in FgSSP‐S4. The *ACTIN* gene was used as the reference. (e) Genomic PCR and RT‐PCR analyses of T3 transgenic wheat lines L2 and L4. The wheat GAPDH gene is amplified using the primers across an intron to distinguish genomic DNA and cDNA. (f) FHB resistance in T2 and T3 transgenic wheat lines. Representative images of infected wheat heads are photographed at 21 dpi, and percentage of infected spikelets are calculated. Data presented are the mean ± SE (*n* ≥ 20). (g) Relative mRNA expression of three target genes of *F. graminearum* during infection of transgenic lines and Yangmai 16 (Y16) at 3 dpi. (h) ICs (as yellow arrows indicate) of *F. graminearum* on wheat lemma from two T3 transgenic lines and Y16 at 3 dpi by scanning electron microscopy (SEM) observation. (i) The average number of ICs in a SEM examination field. (j) DON content of wheat kernels from T3 transgenic wheat lines and Y16 at 35 dpi. (k) Length distribution and abundance of siRNAs targeting *FgSGE1*, *FgSTE12* and *FgPP1*, respectively, in T3 transgenic wheat line L2. Data presented are the mean ± SD (*n* = 3). Different letters indicate a significant difference (*P* < 0.05) according to Student’s *t*‐test

Next, the chimeric RNAi cassette expressed in plant was constructed (Figure 1a) and then bombarded into a FHB‐susceptible wheat cultivar Yangmai 16 (Y16) to generate transgenic plants. Integration and expression of the RNAi cassette were verified by PCR and reverse transcription PCR (RT‐PCR), respectively, in two independent transgenic lines L2 and L4 (Figure 1e). The flowering spikes of transgenic wheat plants were inoculated with single‐floret injection to evaluate their susceptibility against FHB under field condition, where the FHB‐resistant cultivar Sumai 3 was used as a positive control. At 21 days of post‐inoculation (dpi), typical and serious scab symptoms were observed on wheat heads of Y16 (44% infected spikelets), while wheat heads of two transgenic wheat lines showed dramatically attenuated disease symptoms (20.0% to 24.9% infected spikelets), similar to Sumai 3 (Figure 1f). To verify whether FHB resistance in the transgenic wheat lines is caused by *in planta*‐derived silencing of three target genes, we quantified the transcription level of these target genes in *F. graminearum* during infection process on wheat heads of T3 transgenic and control wheat at 3 dpi. Results showed that the relative transcriptional levels of the *FgSGE1*, *FgSTE12* and *FgPP1* were reduced by about 60%–70%, 50%–60% and 25%–35%, respectively, in two transgenic lines, compared to the Y16 control (Figure 1g). These results suggest that host delivered siRNAs might effectively trigger the RNAi in fungal cells and subsequently reduce the transcripts of the target genes during infection. In line with the decreased transcriptional level of *FgSTE12* in infected transgenic wheat heads, the fungal infection structures were dramatically reduced on the inoculated wheat spikelets of L2 and L4 at 3 dpi (Figure 1h‐i). Similarly, decreased expression of *FgSGE1* in *F. graminearum* on tissues of transgenic plants during infection resulted in less DON production in grains harvested from the whole infected spikes, compared with Y16 (Figure 1j). Collectively, the HIGS transgenic wheat plants expressing chimeric *FgSGE1*‐*STE12*‐*PP1* RNAi construct increased resistance to FHB and inhibited DON biosynthesis during the fungal infection.

To further confirm that silencing of the three target genes in infected *F. graminearum* was mediated by homologous siRNAs generated in transgenic wheat plants, small RNA sequencing was performed to identify siRNAs specific to the RNAi cassette in transgenic wheat lines L2 and L4. The sequencing data showed that siRNAs mapping to the *FgSGE1*, *FgSTE12* and *FgPP1* genes were significantly enriched in the two independent transgenic lines, accounting for 1.38% and 3.90% of the total small RNAs detected, respectively, in L2 and L4. The lengths of siRNAs mapped to any of the three genes in transgenic lines L2 and L4 were distributed between 18 and 30 bp, wherein 21 bp was the most abundant, followed by 22‐bp and 24‐bp (Figure 1k). These results demonstrated that *FgSGE1*‐*STE12*‐*PP1* RNAi constructs were successfully processed into siRNA molecules in transgenic wheat plants, and these siRNAs were translocated to fungal cells during infection, thereby reducing the transcript levels of three target genes in the invading hyphae of *F. graminearum*.

In summary, our results suggest that HIGS targeting multiple genes involved in both primary and secondary metabolisms of the fungus is effective and can be used as an alternative approach for developing FHB and mycotoxin resistant crops.

## Conflict of interest

The authors declare no conflict of interest.

## Author contributions

Z.M., X.Z. and Y.C. designed the studies. M.W., L.W. and Y.M. performed experiments. M.W., Y.Z., Z.M. and Y.C. wrote the paper.

## References

[pbi13401-bib-0001] Bai, G.H. and Shaner, G. (2004) Management and resistance in wheat and barley to Fusarium head blight. Annu. Rev. Phytopathol. 42, 135–161. 15283663 10.1146/annurev.phyto.42.040803.140340

[pbi13401-bib-0002] Chen, Y. , Kistler, H.C. and Ma, Z.H. (2019) *Fusarium graminearum* trichothecene mycotoxins: biosynthesis, regulation, and management. Annu. Rev. Phytopathol. 57, 15–39. 30893009 10.1146/annurev-phyto-082718-100318

[pbi13401-bib-0003] Cheng, W. , Song, X.S. , Li, H.P. , Cao, L.H. , Sun, K. , Qiu, X.L. , Xu, Y.B. *et al*. (2015) Host‐induced gene silencing of an essential chitin synthase gene confers durable resistance to Fusarium head blight and seedling blight in wheat. Plant Biotechnol. J. 13, 1335–1345. 25735638 10.1111/pbi.12352

[pbi13401-bib-0004] Gu, Q. , Zhang, C.Q. , Liu, X. and Ma, Z.H. (2015) A transcription factor FgSte12 is required for pathogenicity in *Fusarium graminearum* . Mol. Plant Pathol. 16, 1–13. 24832137 10.1111/mpp.12155 PMC6638345

[pbi13401-bib-0005] Jonkers, W. , Dong, Y.H. , Broz, K. and Kistler, H.C. (2012) The Wor1‐like protein Fgp1 regulates pathogenicity, toxin synthesis and reproduction in the phytopathogenic fungus *Fusarium graminearum* . PLoS Pathog. 8, e1002724. 22693448 10.1371/journal.ppat.1002724 PMC3364952

[pbi13401-bib-0006] Koch, A. , Kumar, N. , Weber, L. , Keller, H. , Imani, J. and Kogel, K.H. (2013) Host‐induced gene silencing of cytochrome P450 lanosterol C14 alpha‐demethylase‐encoding genes confers strong resistance to *Fusarium* species. Proc. Natl. Acad. Sci. USA, 110, 19324–19329. 24218613 10.1073/pnas.1306373110 PMC3845197

[pbi13401-bib-0007] Machado, A.K. , Brown, N.A. , Urban, M. , Kanyuka, K. and Hammond‐Kosack, K.E. (2018) RNAi as an emerging approach to control Fusarium head blight disease and mycotoxin contamination in cereals. Pest Manag. Sci. 74, 790–799. 28967180 10.1002/ps.4748 PMC5873435

[pbi13401-bib-0008] Yun, Y.Z. , Liu, Z.Y. , Yin, Y.N. , Jiang, J.H. , Chen, Y. , Xu, J.R. and Ma, Z.H. (2015) Functional analysis of the *Fusarium graminearum* phosphatome. New Phytol. 207, 119–134. 25758923 10.1111/nph.13374

